# Deletion of *Salmonella enterica* serovar Typhi *tolC* reduces bacterial adhesion and invasion toward host cells

**DOI:** 10.3389/fmicb.2023.1301478

**Published:** 2023-11-03

**Authors:** Ashraf Hussain, Eugene Boon Beng Ong, Prabha Balaram, Asma Ismail, Phua Kia Kien

**Affiliations:** Institute for Research in Molecular Medicine (INFORMM), University Sains Malaysia, Penang, Malaysia

**Keywords:** *Salmonella* Typhi, TolC, Salmonella pathogenicity island 1, invasion, efflux pump protein, adhesion, pathogenesis, antibiotic resistance

## Abstract

**Background:**

*S*. Typhi is a Gram-negative bacterium that causes typhoid fever in humans. Its virulence depends on the TolC outer membrane pump, which expels toxic compounds and antibiotics. However, the role of TolC in the host cell adhesion and invasion by *S*. Typhi is unclear.

**Objective:**

We aimed to investigate how deleting the *tolC* affects the adhesion and invasion of HT-29 epithelial and THP-1 macrophage cells by *S*. Typhi *in vitro*.

**Methods:**

We compared the adhesion and invasion rates of the wild-type and the *tolC* mutant strains of *S*. Typhi using in vitro adhesion and invasion assays. We also measured the expression levels of SPI-1 genes (*invF, sipA*, *sipC*, and *sipD*) using quantitative PCR.

**Results:**

We found that the *tolC* mutant showed a significant reduction in adhesion and invasion compared to the wild-type strain in both cell types. We also observed that the expression of SPI-1 genes was downregulated in the *tolC* mutant.

**Discussion:**

Our results suggest that TolC modulates the expression of SPI-1 genes and facilitates the adhesion and invasion of host cells by *S*. Typhi. Our study provides new insights into the molecular mechanisms of *S*. Typhi pathogenesis and antibiotic resistance. However, our study is limited by the use of *in vitro* models and does not reflect the complex interactions between *S*. Typhi and host cells *in vivo*.

## 1. Introduction

Efflux pumps are present in all major pathogenic bacterial species lineages (Piddock, 2006a,b). In Gram-negative bacteria, this active efflux mainly contributes to the intrinsic resistance to several classes of antibiotics, dyes, and detergents (Li and Nikaido, 2004; Piddock, 2006a,b; Sun et al., 2014). In Gram-negative bacterial pathogens, TolC is an outer membrane efflux pump protein that facilitates efflux and contributes to virulence and pathogenesis (Piddock, 2006a,b). The expression of efflux pumps was observed along with the infection process of Gram-negative pathogens (Fernando and Kumar, 2013). Outer membrane efflux pump proteins (OMPs) have essential functions in the physiology of bacteria, such as adhesion and invasion of the host cell, resistance to host serum, maintenance of the membrane integrity, and passive and active transfer of substances (Tokuda, 2009). Although the biological functions of TolC homologs in several Gram-negative bacteria have already been reported, the role of TolC in *Salmonella enterica* serovar Typhi has not been investigated, especially its role in host cell adhesion and invasion. *S*. Typhi is a human-restricted pathogen that causes typhoid fever.

In this study, the role of *S*. Typhi TolC in the adhesion and invasion of host cells were first investigated using a *tolC* mutant. As the expression of Salmonella pathogenicity island 1 (SPI-1) genes for invasion (*sipA, sipC, sipD*, and *invF*) is required for penetration into host cells (Darwin and Miller, 1999a,b), we also investigated the expression of the invasion genes under SPI-1-inducing conditions.

## 2. Materials and methods

### 2.1. Bacterial strains and plasmids

A *Salmonella enterica* serovar Typhi (*S*. Typhi) strain (a clinical isolate from an acute typhoid fever patient), denoted as ST-WT was used as the wild-type strain in this study.

This study was conducted in accordance with the ethical principles of the Declaration of Helsinki and the EEC directive of 1986. The *Salmonella enterica* serovar Typhi strain was obtained from patients with acute typhoid fever subjects in Hospital Universiti Sains Malaysia (HUSM). The strain was deposited in the Bank of the Institute for Research in Molecular Medicine (INFORMM), Kubang Kerian, Kelantan, Malaysia. The collection and use of the strain were approved by the Committee for Human Ethical Clearance of Universiti Sains Malaysia, Kubang Kerian, Malaysia under ethical clearance number USMKK/PPP/JEPeM [229.3. (03)]. All participants gave their informed consent before enrollment in the study. Bacterial strains and plasmids used in this study are summarized in Table 1. All strains were routinely grown in Luria-Bertani (LB) agar and broth (Hi-media) at 37°C with antibiotics for selection when required.

**Table 1:** Strains and plasmids used in this study.

	Description	Resistance phenotype	Source or reference
Strains			
ST-WT	*S.* Typhi clinical isolate which served as wild type in this study.	None	This study
ST-∆*tolC*	*S.* Typhi TolC mutant, ∆*tolC*::kanR.	KanR	This study
ST-∆*tolC*+	ST-∆*tolC* carrying pKK-tolC.	KanR, AmpR	This study
DH5α	*Escherichia coli* strain for gene cloning and plasmid propagation.		This study
Plasmids			
pKD13	A kanamycin marker-DNA template vector for gene disruption.	KanR	Datsenko and Wanner (2000)
pKD46	Red helper plasmid encoding the λ red recombination system for homologous recombination.	AmpR	Datsenko and Wanner (2000)
pKK-tolC	A plasmid containing *S.* Typhi *tolC* including its native promoter. For complementation in ST-∆*tolC*.	AmpR	This study

### 2.2. Construction of *tolC* mutant

A *tolC* deletion mutant (ST-Δ*tolC*) was constructed using the one-step inactivation of the chromosomal gene method (Baba et al., 2006). The *tolC* gene of ST-WT strain was replaced by inserting the kanamycin resistance gene *aph* (3′)-II genes that confers kanamycin resistance to generate strain ST-Δ*tolC*. A complementation mutant (ST-∆*tolC*+) was also constructed by cloning the *tolC* gene, including its native promoter, into the pKK223-3 plasmid and transformed into ST-Δ*tolC*. Primers for construction of *tolC* mutant summarized in Table 2.

**Table 2:** Primers used in this study to the construction of *tolC* deletion mutant of *S*. Typhi.

Primers set	Primers	Sequence	Description	Reference
1	ST_TolC_Del_Fwd	5′–TTACAAATTGATCAGCGCTAAATACTGCTTCACAACAAGGAATGCAAATGATTCCGGGGATCCGTCGACC–3′	Forward primer for amplification of deletion fragment	This study
	ST_TolC_Del_Rev	5′–CTGATAAACGCAGCGCCAGCGAATAACTTATCAATGCCGGAATGGATTGCTGTAGGCTGGAGCTGCTTCG –3′	Reverse primer for amplification of deletion fragment	
2	1a	5′–GCGACCATCTCCAGCAGC–3′	Upstream check	This study
	1b	5′ – TGCTCTTCGTCCAGATCATC–3′	Internal *Kan^r^* forward check	
3	2a	5′ – GCGAGCACGTACTCGGATGG–3′	Internal *Kan*^r^ reverse check	This study
	2b	5′ – ATGCGGCGGAATAGCAGGAT–3′	Downstream check	
4	H_Fwd	5′ –ACTCAGGCTTCCCGTAACGC–3′	*Salmonella* specific	(Levy et al., 2008)
	H_Red	5′ – GGCTAGTATTGTCCTTATCGG–3′		
5	4a	5′–TTAATGGATCCAGAGAACCTGATGCAAGTT–3′	Internal *tolC* check forward	This study
	4b	5′–GACGGCTCGAGTCAACCGTTCGCATCGCGATA–3′	Internal *tolC* check reverse	
6	*tolC*_F	5′-TTAATGAATTCTTACGCATTGTGCTGCCC	*tolC* complementation forward	This study
	*tolC*_R	5′-GACGGAAGCTTTCAATGCCGGAATGGATT	*tolC* complementation reverse	

### 2.3. Growth of *Salmonella enterica* serovar Typhi strains

The growth of all *S*. Typhi strains was determined by measuring the optical density (OD 595 nm) of bacterial culture using LB broth in a microtiter plate at 37°C as previously described (Sheridan et al., 2013). The sample was placed in a microtiter plate, and bacterial growth was recorded at 2-h time intervals (Multiskan Spectrum, Thermo Scientific). All growth experiments were performed three times, and results were analyzed by plotting average values for each strain on a logarithmic graph with calculated standard deviations to use as error bars. Generation times were also calculated using the logarithmic growth phase of the line graph by the following equation;


$$g=T\;\ln2/\ln\;\left(OD\;\text{end}/OD\;\text{start}\right)$$

(Where g is generation [doubling] time and T is time, ln is Natural log of given value). Values which appeared different to the parent strain were evaluated for significance at defined time points using a student’s ‘*t*’ test. OD end is the optical density at the end of the exponential growth phase, OD start is the optical density at the start of the exponential growth phase.

### 2.4. Efflux activity assay

Efflux activity was evaluated with the ethidium bromide agar screening method (Martins et al., 2006). Overnight cultures of all strains were swabbed onto LB agar plates containing 0.5 and 1 mg/L of ethidium bromide. The plates were incubated at 37°C for 16 h, and the fluorescence intensity associated with efflux pump function in the bacterial mass was photographed.

### 2.5. Adhesion and invasion assays

The adhesion and invasion of the *S*. Typhi strains were tested using *in vitro* adhesion and invasion assays with THP-1-derived human macrophages and HT-29 human epithelial cells according to the methods of Dibb-Fuller et al. (1999) and Buckley et al. (2006) with some modification. *S*. Typhi cells were grown overnight in 10 mL LB broth at 37°C. Cell monolayers were grown to confluence in 6-well plates, and the cells were then infected for 2 h at an infection multiplicity of 50. For the adhesion assay, cells were gently washed six times with phosphate-buffered saline (PBS) (pH 7.3) and then disrupted with 1 mL distilled water (Roche et al., 2005). First, all viable bacteria (intra- and extracellular) were counted as colony-forming units after plating serial dilutions with PBS. Then, the entry of *S*. Typhi into HT-29 cells and THP-1 macrophages was quantified by the invasion assay (also called gentamicin protection assay) as previously described (Amy et al., 2004) to quantify intracellular bacteria. All the bacterial strains tested have similar gentamicin susceptibility (data not shown). To calculate the bacterial adhesion, we subtracted the cfu/ml of the invaded bacteria from the cfu/ml of the total viable bacteria that were either adherent to the cell surface or inside the cell after washing step. Results are expressed as cfu/ml of adherent and invasive bacteria compared to the ST-WT strain.

All quantitative invasion assays were performed separately for each strain in triplicates. ST-∆*tolC* and ST-∆*tolC*+ were compared with the ST-WT reference strain using Student’s *t*-test. Each strain’s overall mean cfu/ml was calculated for each biological replicate.

### 2.6. Reverse transcription PCR of SPI-1 gene expression

Reverse transcription polymerase chain reaction (RT-PCR) was performed to measure the transcription of the invasion-related genes of *S*. Typhi, according to the study of Webber et al. (2009). Bacteria were grown in LB broth until the mid-log phase (OD_600_ of 0.6) containing 0.3 M NaCl for SPI-1-inducing conditions (Arricau et al., 1998). Total bacterial RNA was obtained from the bacteria by using the RNeasy mini kit (Qiagen) according to the manufacturer’s recommendation. Possible DNA contamination was removed by treating with DNase I (Sigma). The absence of DNA contamination was tested by PCR amplification using total RNA as a template and primers specific for the *recA* housekeeping gene (Wong et al., 2013). The purity and concentration of RNA were determined by measuring the optical density at 230, 260, and 280 nm before use (A260/280 ranged from 1.8 to 2.0). The quality of the RNA was assessed by gel electrophoresis and ethidium bromide staining.

Real-time RT-PCR was performed to measure the transcriptional level of the *invF, sipA, sipC*, and *sipD*, and the housekeeping gene *recA* was used as a control (Wong et al., 2013). Specific primer sequences were designed for each gene using the Primer3 software program (Table 3). First, 1 μg of DNase-treated total RNA from at least three independent cultures were reverse transcribed using random hexamers and Superscript III 1st Strand Kit (Invitrogen, Cat #18080-051). Then, the amplification was performed using the QuantiFast SYBR Green PCR Kit (Qiagen) on an Applied Biosystems^™^ 7500 Real-Time PCR System according to the manufacturer’s instructions. The amplification of *recA* for target gene expression normalization was conducted simultaneously with the amplification of targeted genes. Fold-change of expression level for a target gene was determined using the comparative Ct approach, whereby the Ct value of the target gene in each sample was normalized to *recA*, and the relative expression level of the target gene and the fold-change in gene expression were calculated (Livak and Schmittgen, 2001). The expression of a target gene was presented as the fold change relative to the ST-WT strain. Data were obtained in three separate experiments with three technical replicates. All results were analyzed by the Student’s *t*-test, and *p* values of <0.05 indicate significance.

**Table 3:** Oligonucleotide sequences for the amplification of selected SPI-1 genes in RT-PCR.

Target genes	Oligonucleotide sequence		Amplicon size
*sipA*	Forward	5′-CGCGTGTGGATTCGACTACG-3′	127 bp
	Reverse	5′-GAGTTGGTCACAGCCTCTGC-3′	
*sipC*	Forward	5′-CAGTGACCTGGGGTTGAGTC-3′	135 bp
	Reverse	5′-GCCAGGGCATTCAAATCCTG-3′	
*sipD*	Forward	5′-TTCTCCTCATCCGGGGATCG-3′	100 bp
	Reverse	5′-GCCGCGATGTTCTGTGGTAG-3′	
*invF*	Forward	5′-GTCGTTTGTGCAGCAGAGC-3′	107 bp
	Reverse	5′-GGTGATGTTCTCGTGGCCTT-3′	
*recA*	Forward	5′-CAGGCCGAGTTCCAGATCCT-3′	120 bp
	Reverse	5′-CTCGCCGTTGTAGCTGTACC-3′	

## 3. Results

### 3.1. The growth of the *tolC* mutant was defective

**Figure 1:**
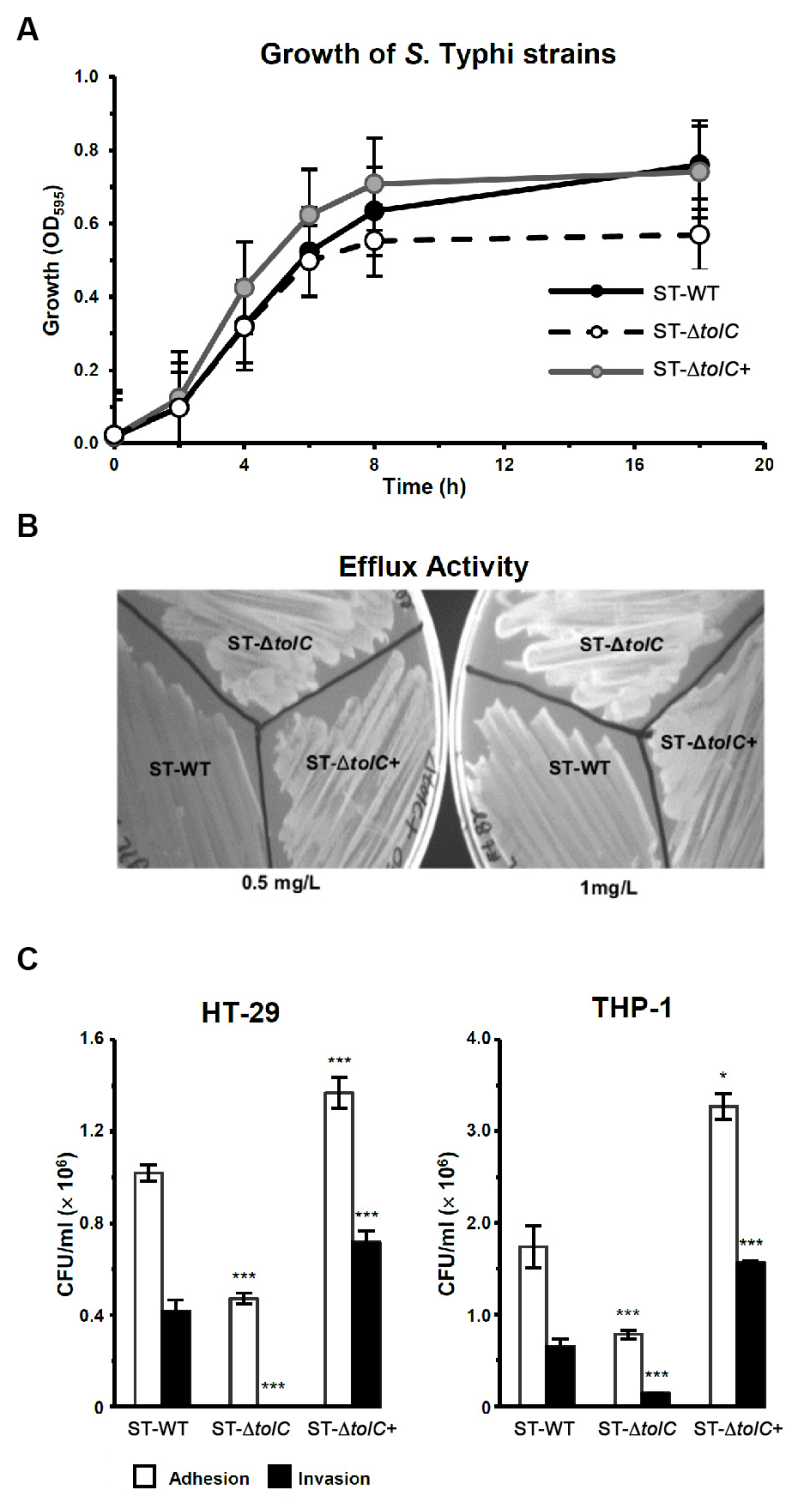
Deletion of *S*. Typhi *tolc* affects bacterial growth, efflux, adhesion, and invasion. **(A)** *S*. Typhi strains ST-WT, ST-∆*tolC*, and ST-∆*tolC+* were grown at 37°C, and their OD was recorded for up to 18 h. **(B)** Evaluation of the efflux activity of *S*. Typhi strains. Strains were cultured on an LB plate containing 0.5 and 1.0 mg/L ethidium bromide overnight at 37°C and observed under UV light. **(C)** Evaluation of the adhesion and invasion activity of *S*. Typhi strains on HT-29 and THP-1 cells. Data are displayed as the mean of at least three separate experiments performed in triplicate ± standard deviation. Values returning a *p* value of ≤0.001 from a Student’s *t*-test comparing ST-∆*tolC* and ST-∆*tolC+* strains to the ST-WT strain. The asterisks above the bars represent the significance of the *t*-test, *** < 0.001.

To evaluate the fitness of the strains, we observed the growth of the strains by OD measurement. The ST-WT and ST-Δ*tolC* had similar doubling times of 112 and 116 min (*p = 0.55*), respectively (Figure 1A), while the ST-∆*tolC*+ had a shorter doubling time of ~80 min (*p = 0.004*). The ST-Δ*tolC* and ST-∆*tolC*+ strains arrived at the stationary stage at 8 h; however, ST-WT continued growing at a slower rate with an OD595 reading of ~0.63, ~0.70, and ~ 0.55, respectively. The growth of the bacteria reached a stationary phase after 18 h of incubation. The OD595 of the ST-WT and the ST-∆*tolC*+ strain was similar, with mean values of ~ 0.76 and ~ 0.74, respectively (*p* = 0.20). However, the OD595 of the ST-Δ*tolC* strain was significantly lower, with a mean value of ~0.56 (*p* < 0.05). Based on the growth curves (Figure 1A), it is deduced that the ST-Δ*tolC* cell growth defect may be due to the lack of the TolC efflux pump. In contrast, the growth defect of the mutant was rescued by the presence of the plasmid carrying *tolC* (ST-∆*tolC*+) complement strain.

### 3.2. The efflux activity of the *tolC* mutant was impaired

To evaluate the efflux activity of the ST-Δ*tolC* strain, all strains (ST-WT and ST-Δ*tolC*+ as controls) were streaked on media containing ethidium bromide. After incubation, the plates were observed under UV light. The ST-WT appeared to fluoresce the least, followed by ST-Δ*tolC+* and ST-Δ*tolC* (Figure 1B). Fluorescence intensity from the cells is inversely proportional to efflux pump function, indicating pump functionality in ST-WT and impairment in ST-Δ*tolC* (at both lower and higher ethidium bromide concentrations). Even though ST-Δ*tolC+* was expected to have efflux activity similar to ST-WT, the strain’s efflux activity was affected (Figure 1B), suggesting that the TolC expressed from the plasmid could be different from the chromosome expressed protein in ST-WT.

### 3.3. The adhesion and invasion of *tolC* mutant were reduced

To investigate the adhesion and invasion abilities of the strains, the bacterial cells were added to the human intestine epithelial HT-29 and macrophage THP-1 cells. Declines in the adhesion and invasion abilities of ST-∆*tolC* were observed when the bacterial cell interacted with both host cell types *in vitro* (Figure 1C). The adhesion efficiency of ST-∆*tolC* was reduced by ~55% in both host cell types compared to ST-WT. The significant loss of invasion is most likely due to the loss of adhesion capability in ST-∆*tolC* because adhesion is a crucial step preceding invasion. Interestingly, the ST-∆*tolC+* strain showed increased adhesion and invasion in both host cell types. We speculate that the presence of multiple copies of *tolC* on the plasmids likely contributed to this phenomenon.

### 3.4. Invasion-related genes were downregulated in the *tolC* mutant

**Figure 2:**
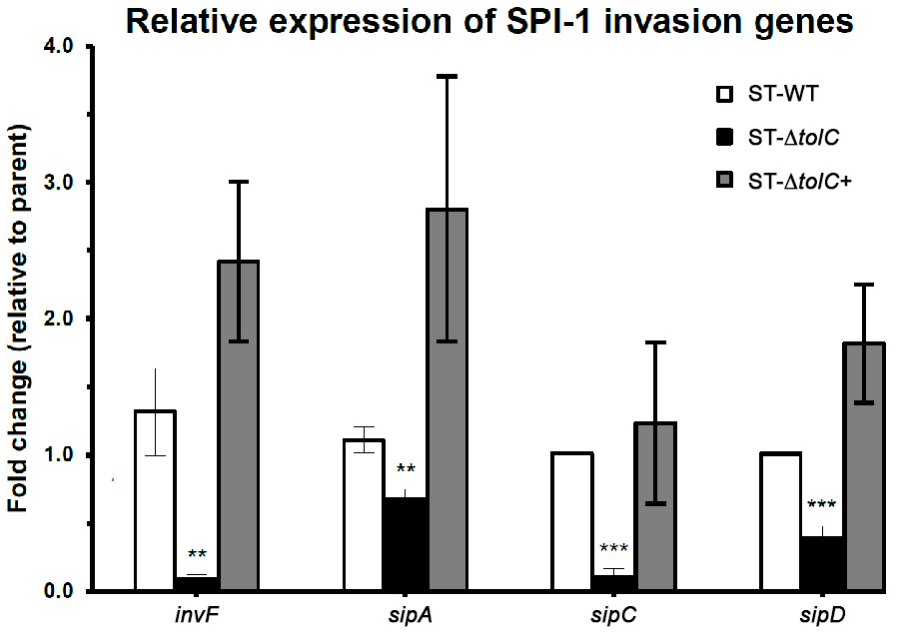
The relative expression of selected invasion-related SPI-1 genes in *S*. Typhi strains. The strains were cultured in SPI-1-inducing condition (0.3 M NaCl), and RT-PCR was performed to quantify relative gene expression. The expression of a target gene was presented as the fold change relative to the ST-WT strain used as a control in this study. White bars indicate ST-WT, black bars ST-∆*tolC*, and gray bars for ST-∆*tolC*+. Bars indicate the messenger RNA fold-changes observed in ST-∆*tolC*, and ST-∆*tolC+* compared to their ST-WT reference strain with +/− standard deviation and the mean of three independent experiments. The asterisks above bars represent significance from a Student’s *t*-test, ** < 0.01, *** < 0.001.

To evaluate the direct efflux function of TolC and the indirect role of the presence of *tolC* on the expression of SPI-1 TTSS-1 genes, we cultured the strains in the SPI-1-inducing condition (0.3 M NaCl). We performed RT-PCR to quantify relative gene expression. The mRNA expression of invasion-related genes such as *sipA, sipC, sipD*, and *invF* was significantly reduced in ST-Δ*tolC* when compared with the ST-WT reference strain that was used as control; indeed, the transcripts of these genes revealed that transcriptions of *invF, sipA, sipC*, and *sipD* genes were decreased, 15–fold, 1.6–fold, 9.6–fold, and 2.5–fold, respectively, in the ST-Δ*tolC* when compared with the ST-WT reference strain. Although the complementation ST-∆*tolC*+ strain significantly increased the transcriptions of these genes, *sipA* (2.8-fold), *invF* (2.4-fold), *sipC* (1.2 -fold), and *sipD* (1.8-fold), when compared with ST-WT (Figure 2).

## 4. Discussion

The molecular mechanisms of *Salmonella* host cell entry and its intracellular survival have been widely investigated in the past few decades, and key bacterial invasion factors (e.g., SPI-1 TTSS-1) have been identified (Kaufmann et al., 2001; Ribet and Cossart, 2015). While previous studies have reported TolC-related functions in *S*. Typhimurium virulence (i.e., colonization, persistence, adhesion, and invasion) (Buckley et al., 2006; Nishino et al., 2006), no studies have been reported for the role of TolC in *S*. Typhi. In this study, we hypothesized that the *S*. Typhi TolC would similarly play an essential role in bacterial adhesion and invasion during the infection of human cells and also investigated whether the lack of TolC will affect SPI-1 gene expression known to be upregulated during the bacterial invasion.

We first constructed a *tolC* mutant, and the analysis of its growth showed that during the log phase, the growth profiles of ST-Δ*tolC* were similar to the ST-WT strain; however, it reached the stationary phase earlier and at a lower OD (Figure 1A). One plausible explanation is that intracellular waste and metabolites accumulated due to impaired efflux, and this internal toxicity affects cell viability, thus leading to an earlier stationary phase and lower cell density. Alternatively, it may be due to the altered expression of genes involved in stress response, virulence, or metabolism that are regulated by *tolC* or its associated operons. Further studies are needed to elucidate the molecular mechanisms underlying the growth defect of ST-Δ*tolC* in the stationary phase and its implications for pathogenesis.

Our results are consistent with some previous reports on *tolC* mutants of other bacterial species. For example, Virlogeux-Payant et al. (2008) showed that a *tolC* mutant of *Salmonella Typhimurium* had a comparable growth rate to the wild-type strain based on the growth curve, but they did not report the growth characteristics of the mutant in the stationary phase. Webber et al. (2009) reported that a *tolC* mutant of *Salmonella Typhimurium* did not show a growth defect in the log phase compared to the wild-type strain. However, Santos et al. (2010) reported that a *tolC* mutant of *Sinorhizobium meliloti* had a similar growth rate to the wild-type strain for the first 8 h of growth, but then showed a reduced growth rate and decreased biomass formation (Santos et al., 2010). Similarly, in our study, ST-Δ*tolC* had reduced growth after 8 h compared to ST-WT.

The ST-Δ*tolC* also showed hyper-susceptibility to detergents and antibiotics (results shown in Supplementary material) as demonstrated by its weaker ability to efflux ethidium bromide, unlike the ST-WT strain (Figure 1B).

In the cell adhesion and invasion assay, the ST-∆*tolC* was significantly less invasive than the ST-WT reference strain in both the epithelium (HT-29) and macrophage (THP-1) cells. The invasion of ST-∆*tolC* was removed entirely in HT-29 cells and was approximately 20% in THP-1 cells, possibly due to the phagocytic activity of the macrophages. As predicted, the adhesion and invasion activity were restored to a higher level in the ST-∆*tolC+* strain in both HT-29 and THP-1 (Figure 1C). These findings are consistent with the study on an *S*. Typhimurium *tolC* mutant where it was also reported to be less adherent to epithelial cells than its WT parent and that TolC is crucial for virulence-related phenotypes such as adhesion and invasion to the host cells (Buckley et al., 2006; Virlogeux-Payant et al., 2008).

The growth curves and invasion assays resulting from this report are consistent with our previous preliminary study that was done with other *S*. Typhi strains (Hussain et al., 2016).

Next, we wanted to see if the suppressed invasion activity of ST-Δ*tolC* was also linked to the TTSS-1 system, which is primarily associated with invasion (Galan, 2001). Using RT-PCR, we found lower expression of invasion-related genes of the TTSS-1 (*invF*, *sipA, sipC*, and *sipD*) (Figure 2). The transcription of *invF*, a transcriptional regulator which activates the transcription of other SPI-1 genes (Darwin and Miller, 1999a,b), was downregulated by fifteen-fold in the ST-Δ*tolC* compared to the ST-WT. When the *tolC* was complemented, the expressions of the SPI-1 genes in the ST-Δ*tolC*+ strain were higher than ST-WT. The higher gene expression levels of ST-∆*tolC*+ also reflect the strain’s higher adhesion and invasion activities (Figure 1C). In *S*. Typhimurium, deletion of another gene *invH*, located downstream of *invF*, was shown to partially impair the secretion of Sip effector proteins (SipABCD) (Pati et al., 2013).

The reduced expressions of the invasion-related genes are likely mediated by the lowered expression of *invF* in the ST-Δ*tolC*. This downregulation is in agreement with the observation in *S*. Typhimurium (*tolC::aph*), where the *invF* was significantly downregulated in the *tolC* mutant (Webber et al., 2009). The *invF* is a positive regulator of SPI-1 that has been confirmed to be essential for the expression of several SPI-1 genes (Darwin and Miller, 1999a,b).

## 5. Conclusion

In this study, we demonstrated that TolC-dependent efflux systems play a vital role in the adhesion and invasion of *S*. Typhi to host cells, which are key steps in the pathogenesis of typhoid fever. We also revealed that TolC influences the expression of SPI-1 genes, which encode the type III secretion system (TTSS-1) that mediates the invasion process. Our findings suggest that TolC is a multifunctional protein that modulates both the efflux activity and the virulence gene expression of *S*. Typhi. However, the exact mechanism by which TolC regulates SPI-1 genes remains unknown and requires further investigation. Moreover, the potential of TolC as a target for developing novel anti-virulence strategies against *S*. Typhi needs to be explored in future studies.

## Data availability statement

The original contributions presented in the study are included in the article/Supplementary material, further inquiries can be directed to the corresponding authors.

## Ethics statement

The studies involving humans were approved by ethical clearance number USMKK/PPP/JEPeM [229.3. (03)]. The studies were conducted in accordance with the local legislation and institutional requirements. Written informed consent for participation in this study was provided by the participants’ legal guardians/next of kin.

## Author contributions

AH: Conceptualization, Data curation, Formal analysis, Investigation, Writing – original draft, Writing – review & editing. EO: Funding acquisition, Project administration, Resources, Supervision, Validation, Visualization, Writing – review & editing. PB: Conceptualization, Formal analysis, Funding acquisition, Project administration, Resources, Supervision, Validation, Visualization, Writing – review & editing. AI: Funding acquisition, Project administration, Resources, Supervision, Writing – review & editing. PK: Funding acquisition, Project administration, Resources, Supervision, Validation, Visualization, Writing – review & editing.

## References

[ref1] Amy M. Velge P. Senocq D. Bottreau E. Mompart F. Virlogeux-Payant I. (2004). Identification of a new *Salmonella enterica* serovar Enteritidis locus involved in cell invasion and in the colonisation of chicks. Res. Microbiol. 155, 543–552. doi: 10.1016/j.resmic.2004.03.005, PMID: 15313254

[ref2] Arricau N. Hermant D. Waxin H. Ecobichon C. Duffey P. S. Popoff M. Y. (1998). The RcsB-RcsC regulatory system of *Salmonella typhi* differentially modulates the expression of invasion proteins, flagellin and Vi antigen in response to osmolarity. Mol. Microbiol. 29, 835–850. doi: 10.1046/j.1365-2958.1998.00976.x, PMID: 9723922

[ref3] Baba T. Ara T. Hasegawa M. Takai Y. Okumura Y. Baba M.. (2006). Construction of *Escherichia coli* K-12 in-frame, single-gene knockout mutants: the Keio collection. Mol. Syst. Biol. 2:2006.0008. doi: 10.1038/msb4100050 PMC1681482 16738554

[ref4] Buckley A. M. Webber M. A. Cooles S. Randall L. P. La Ragione R. M. Woodward M. J.. (2006). The AcrAB-TolC efflux system of *Salmonella enterica* serovar Typhimurium plays a role in pathogenesis. Cell. Microbiol. 8, 847–856. doi: 10.1111/j.1462-5822.2005.00671.x, PMID: 16611233

[ref5] Darwin K. H. Miller V. L. (1999a). InvF is required for expression of genes encoding proteins secreted by the SPI1 type III secretion apparatus in *Salmonella typhi*murium. J. Bacteriol. 181, 4949–4954. doi: 10.1128/JB.181.16.4949-4954.1999, PMID: 10438766 PMC93983

[ref6] Darwin K. H. Miller V. L. (1999b). Molecular basis of the interaction of Salmonella with the intestinal mucosa. Clin. Microbiol. Rev. 12, 405–428. doi: 10.1128/CMR.12.3.405, PMID: 10398673 PMC100246

[ref9001] Datsenko K. A. Wanner B. L. (2000). One-step inactivation of chromosomal genes in Escherichia coli K-12 using PCR products. Proc. Natl. Acad. Sci. U S A, 97, 640–6645. doi: 10.1073/pnas.120163297 10829079 PMC18686

[ref7] Dibb-Fuller M. P. Allen-Vercoe E. Thorns C. J. Woodward M. J. (1999). Fimbriae- and flagella-mediated association with and invasion of cultured epithelial cells by *Salmonella enteritidis*. Microbiology 145, 1023–1031. 10376817 10.1099/13500872-145-5-1023

[ref8] Fernando D. M. Kumar A. (2013). Resistance-nodulation-division multidrug efflux pumps in gram-negative bacteria: role in virulence. Antibiotics 2, 163–181. doi: 10.3390/antibiotics2010163, PMID: 27029297 PMC4790303

[ref9] Galan J. E. (2001). Salmonella interactions with host cells: type III secretion at work. Annu. Rev. Cell Dev. Biol. 17, 53–86. doi: 10.1146/annurev.cellbio.17.1.53, PMID: 11687484

[ref9002] Hussain A. Ong E. B. B. Phei P. S. C. Hossain K. Balaram P. Ismail A.. (2016). Role of TolC in virulence of salmonella enterica serovar Typhi. J. Pure Appl. Microbiol. 10:887.

[ref10] Kaufmann S. H. Raupach B. Finlay B. B. (2001). Introduction: microbiology and immunology: lessons learned from Salmonella. Microbes Infect. 3, 1177–1181. doi: 10.1016/S1286-4579(01)01498-8, PMID: 11755405

[ref9003] Levy H. Diallo S. Tennant S. M. Livio S. Sow S. O. Tapia M.. (2008). PCR method to identify Salmonella enterica serovars Typhi, Paratyphi A, and Paratyphi B among Salmonella Isolates from the blood of patients with clinical enteric fever. J. Clin. Microbiol. 46, 1861–1866. doi: 10.1128/jcm.00109-08 18367574 PMC2395068

[ref11] Li X. Z. Nikaido H. (2004). Efflux-mediated drug resistance in bacteria. Drugs 64, 159–204. doi: 10.2165/00003495-200464020-00004, PMID: 14717618

[ref12] Livak K. J. Schmittgen T. D. (2001). Analysis of relative gene expression data using real-time quantitative PCR and the 2(-Delta Delta C(T)) method. Methods 25, 402–408. doi: 10.1006/meth.2001.1262, PMID: 11846609

[ref13] Martins M. Santos B. Martins A. Viveiros M. Couto I. Cruz A.. (2006). An instrument-free method for the demonstration of efflux pump activity of bacteria. In Vivo 20, 657–664. PMID: 17091774

[ref14] Nishino K. Latifi T. Groisman E. A. (2006). Virulence and drug resistance roles of multidrug efflux systems of *Salmonella enterica* serovar Typhimurium. Mol. Microbiol. 59, 126–141. doi: 10.1111/j.1365-2958.2005.04940.x, PMID: 16359323

[ref15] Pati N. B. Vishwakarma V. Jaiswal S. Periaswamy B. Hardt W. D. Suar M. (2013). Deletion of invH gene in *Salmonella enterica* serovar Typhimurium limits the secretion of Sip effector proteins. Microbes Infect. 15, 66–73. doi: 10.1016/j.micinf.2012.10.014, PMID: 23159244

[ref16] Piddock L. J. (2006a). Clinically relevant chromosomally encoded multidrug resistance efflux pumps in bacteria. Clin. Microbiol. Rev. 19, 382–402. doi: 10.1128/CMR.19.2.382-402.2006, PMID: 16614254 PMC1471989

[ref17] Piddock L. J. (2006b). Multidrug-resistance efflux pumps - not just for resistance. Nat. Rev. Microbiol. 4, 629–636. doi: 10.1038/nrmicro1464, PMID: 16845433

[ref18] Ribet D. Cossart P. (2015). How bacterial pathogens colonize their hosts and invade deeper tissues. Microbes Infect. 17, 173–183. doi: 10.1016/j.micinf.2015.01.004, PMID: 25637951

[ref19] Roche S. M. Gracieux P. Milohanic E. Albert I. Virlogeux-Payant I. Temoin S.. (2005). Investigation of specific substitutions in virulence genes characterizing phenotypic groups of low-virulence field strains of *Listeria monocytogenes*. Appl. Environ. Microbiol. 71, 6039–6048. doi: 10.1128/AEM.71.10.6039-6048.2005, PMID: 16204519 PMC1265998

[ref20] Santos M. R. Cosme A. M. Becker J. D. Medeiros J. M. Mata M. F. Moreira L. M. (2010). Absence of functional TolC protein causes increased stress response gene expression in *Sinorhizobium meliloti*. BMC Microbiol. 10:180. doi: 10.1186/1471-2180-10-180 20573193 PMC2912261

[ref21] Sheridan A. Lenahan M. Condell O. Bonilla-Santiago R. Sergeant K. Renaut J.. (2013). Proteomic and phenotypic analysis of triclosan tolerant verocytotoxigenic *Escherichia coli* O157: H19. J. Proteome 80, 78–90. doi: 10.1016/j.jprot.2012.12.025, PMID: 23313217

[ref22] Sun J. Deng Z. Yan A. (2014). Bacterial multidrug efflux pumps: mechanisms, physiology and pharmacological exploitations. Biochem. Biophys. Res. Commun. 453, 254–267. doi: 10.1016/j.bbrc.2014.05.090, PMID: 24878531

[ref23] Tokuda H. (2009). Biogenesis of outer membranes in gram-negative bacteria. Biosci. Biotechnol. Biochem. 73, 465–473. doi: 10.1271/bbb.80778 19270402

[ref24] Virlogeux-Payant I. Baucheron S. Pelet J. Trotereau J. Bottreau E. Velge P.. (2008). TolC, but not AcrB, is involved in the invasiveness of multidrug-resistant *Salmonella enterica* serovar typhimurium by increasing type III secretion system-1 expression. Int. J. Med. Microbiol. 298, 561–569. doi: 10.1016/j.ijmm.2007.12.006, PMID: 18272427

[ref25] Webber M. A. Bailey A. M. Blair J. M. A. Morgan E. Stevens M. P. Hinton J. C. D.. (2009). The global consequence of disruption of the AcrAB-TolC efflux pump in *Salmonella enterica* includes reduced expression of SPI-1 and other attributes required to infect the host. J. Bacteriol. 191, 4276–4285. doi: 10.1128/JB.00363-09, PMID: 19411325 PMC2698494

[ref26] Wong V. K. Pickard D. J. Barquist L. Sivaraman K. Page A. J. Hart P. J.. (2013). Characterization of the yehUT two-component regulatory system of *Salmonella enterica* Serovar Typhi and Typhimurium. PLoS One 8:e84567. doi: 10.1371/journal.pone.0084567, PMID: 24386394 PMC3875573

