# Supplementary material for: Deletion of Salmonella enterica serovar Typhi tolC reduces bacterial adhesion and invasion toward host cells
Source: Front Microbiol. 2023 Nov 3;14:1301478. doi: 10.3389/fmicb.2023.1301478 (PMC10655110; doi:10.3389/fmicb.2023.1301478)
Supplement: Supplementary file 2 [file Data_Sheet_1.pdf]

Verification of the *tolC* gene deletion in *S*. Typhi by sequencing

To verify insertion of the kan^r in the correct location, the PCR amplicon that produced using primers annealed in the *tolC* and annealed in the kan^r were sequenced (Table 2 , PCRs 6 and 7). The sequence was aligned with the predicted sequence for a *tol*C mutant. The sequence from the candidates was homologous to the predicted sequence that indicates the kan^r gene had inserted at the correct location between base pairs 3363 and 3365.

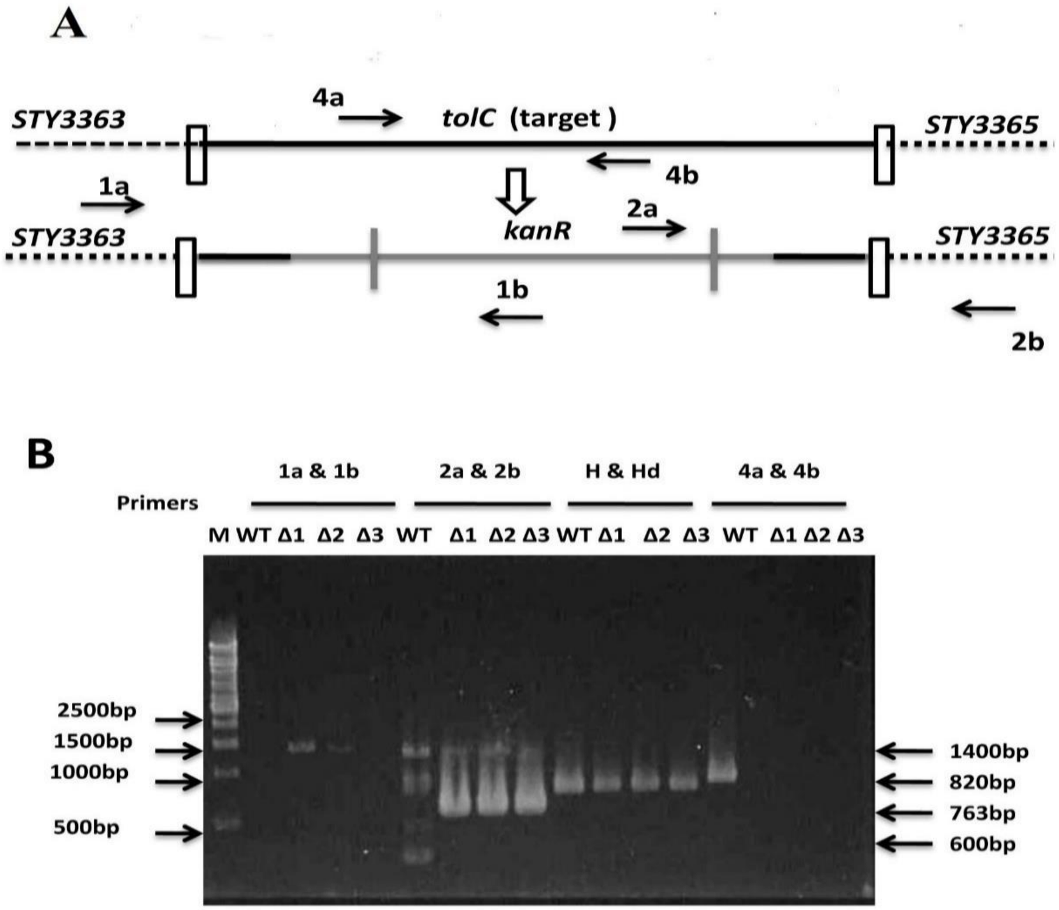

Figure S1: Colony PCR for confirmation of deletion *tolC* from the *S*. Typhi genome.

(A). A schematic representation of primers positions for colony PCR. **(B)**. Gel picture showing the detection of PCR products. Lane M - Molecular weight marker VC 1kb DNA sizing ladder, lane WT - wild-type strain, lane Δ1, Δ2, Δ3 of mutants with 1a, 1b primer set for amplification of 1400 bp band detected only in mutants (Lane, Δ1, Δ2, Δ3) and absent in wild-type (Lane WT). Lane WT - wild-type strain, lane, Δ1, Δ2, Δ3 of mutants with 2a, 2b primer set for amplification of 600 bp band only present in mutants (Lane, Δ1, Δ2, Δ3) and absent in wild-type (Lane WT). *S*. Typhi-specific H, Hd primer set are showing 763 bp amplification in wild-type (Lane WT) and mutants (Lane Δ1, Δ2, Δ3). The *tolC* specific internal primer set, 4a, and 4b are showing 820 bp amplification in wild-type (Lane WT) while 820 bp band is absent in mutant’s strains (Lane, Δ1, Δ2, Δ3).

Table S1 : Verification of the *tolC* deletion by colony PCR

PCR primer code (primer set)	Description	Lane	Predicted fragment size (bp)	Actual fragment size (bp)
1a, 1b	Internal *aph* Check	WT	0	0
		Δ1	1400	1450
		Δ2	1400	14 50
		Δ3	1400	1450
2a, 2b	Internal *aph* check	WT	0	0
		Δ1	600	600
		Δ2	600	600
		Δ3	600	600
H, Hd	*S*. Typhi-specific	WT	763	763
		Δ1	763	763
		Δ2	763	763
		Δ3	763	763
4a,4b	Internal *tolC* Check	WT	820	820
		Δ1	0	0
		Δ2	0	0
		Δ3	0	0

Verification of *S*. Typhi strain by PCR

Finally, additional PCR was done with *Salmonella enterica* serovar Typhi-specific primers and primer set, 2a, and 2b, to confirmed the *tolC* deletion in *S*. Typhi strain. The upstream structure of the *tolC* deletion mutant was confirmed by amplification of 1400 bp band with primer set, 1a, and 1b. *S*. Typhi was confirmed by amplification of 763 bp band with *S*. Typhi-specific primers in both ST-wild-type and ST-Δ*tolC*

strains. PCR products were electrophoresed and visualized on the image analyzer (Alpha Innotech). PCR products sizes from all three candidates were like the predicted size for *Salmonella enterica* serovar Typhi.(Figure 1,2). The Δ2 was selected for further experiments in this study.

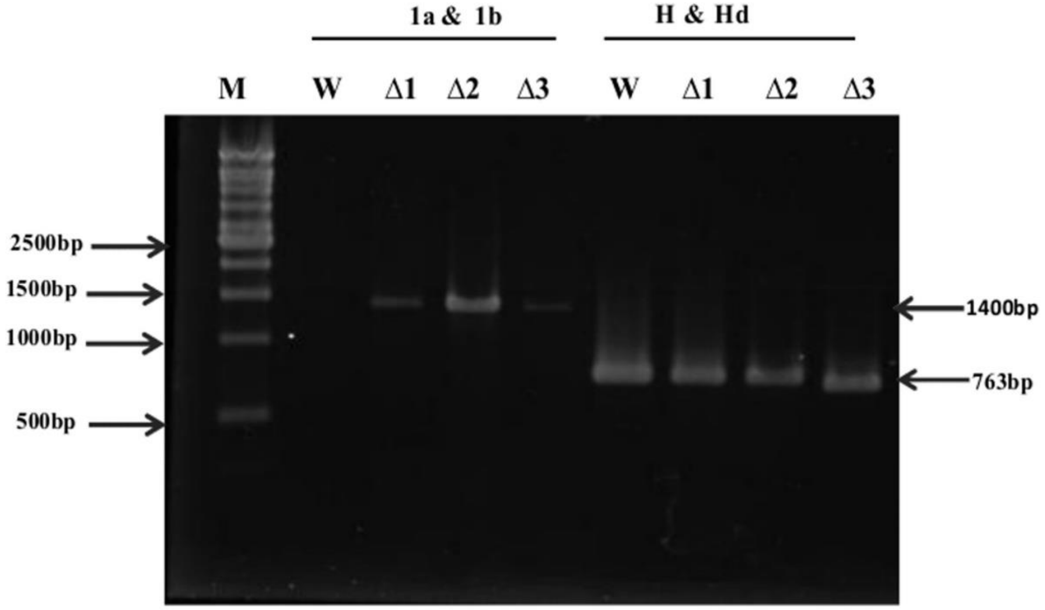

Figure S2 : Agarose gel of colony PCR to confirmation of the *tolC* deletion in *S*. Typhi strain.

Colony PCR with *S*. Typhi-specific primer set and primer set 1a.1b. Lane M marker VC 1kb DNA sizing ladder. Lane W- wild-type strain, lane Δ1, Δ2, Δ3 mutants with 1a, 1b primer set for amplification of 1400 bp band that only present in mutants (Lane Δ1, Δ2, Δ3) and absent in wild-type. *S*. Typhi-specific primer set showing 763 bp amplification in W, Δ1, Δ2, Δ3, lanes. The Δ2 was selected for further experiments in this study

Sensitivity on SDS and kanamycin

S. Typh ST-Δ*tolC* and ST-Δ*tolC*+ strains can grow on LB agar plate containing 30 μg/mL kanamycin, but ST-wild-type was unable to grow because of the absence of kanamycin resistance *kan*^{r} (Figure 3S).

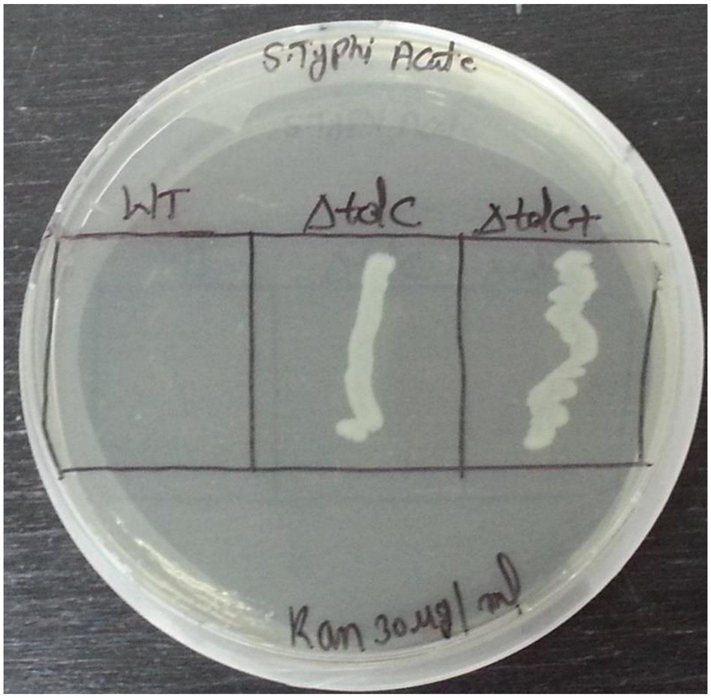

Figure S3: Confirmation of *tolC* deletion by culture on 30 μg/mL kanamycin LA plate.

Wild-Type (WT) was unable to grow, but Δ*tolC* (Δ*tolC*) and complementation strains Δ*tolC*+ (Δ*tolC*+) could grow.

Confirmation of *tolC* deletion on LA plate containing 0.01 % SDS, The ST-Δ*tolC* was unable to grow on 0.01 % SDS while the wild-typea nd complement strains Δ*tolC*+ could grow on the presence of 0.01 % SDS (Figure 4).

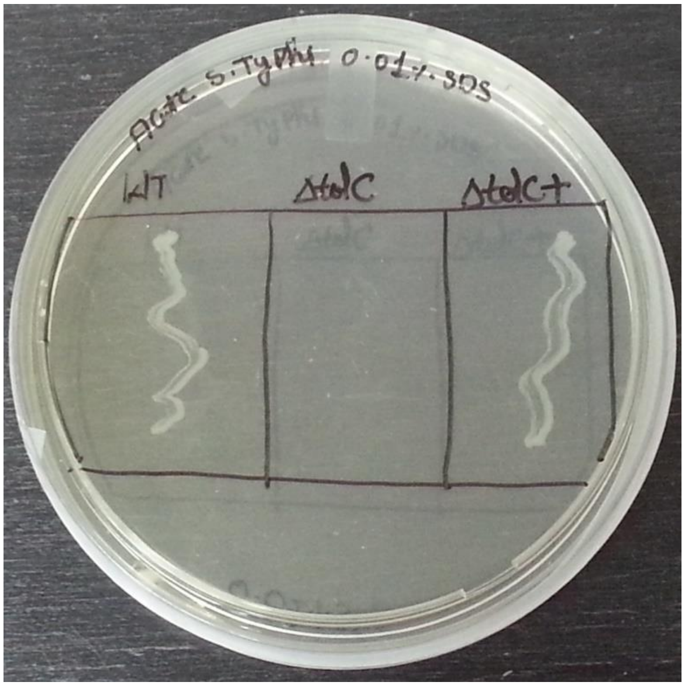

Figure S4: Confirmation of *tolC* deletion by culture on 0.01 % SDS containing LA plate.

Wild-Type B3952/07 (WT) and complement B3952/07Δ*tolC*+ (Δ*tolC+)* strains could grow, but B3952/07Δ*tolC* (Δ*tolC*) was unable to grow on 0.01 % SDS containing LA plate.

Complementation of in the *tolC* mutant

Complementation of ST-Δ*tolC* restored the wild-type phenotype (Figure 3 and Figure 4).

Determination of the minimum inhibitory concentration (MIC)

The MIC of antibiotics (chloramphenicol, tetracycline), detergents (SDS, deoxycholate) were determined for all three bacterial strains (ST-Wild-type, ST-Δ*tolC*, and ST-Δ*tolC*+) by following the BSEN ISO: 20776-1 (2006) protocol. In brief, 50 µL of the appropriate broth was added to wells 2-12 of a 96 wells of microtiter plate. A 50 µL of the chosen biocide (SDS, deoxycholate, antibiotic) was then two-fold serial dilutions across wells 1-11. Columns 12 were control columns without biocide (SDS and deoxycholate concentration range were 1 – 0.0009 %, for antibiotics, 256 µg/mL concentration start from first well), as shown in Figure 5. Suspensions of all bacterial strains were standardized to 1 x 10^8 CFU/mL. A 50 µL of an individual strain was then added to all wells in three rows of the plate (i.e. in triplicate). The plate was then covered with a sterile lid and incubated for 24 hours at 37°C with shaking at 150 rpm. The MIC was the lowest concentration where bacterial growth was not observed in the microtiter plate wells. This was observed visually.

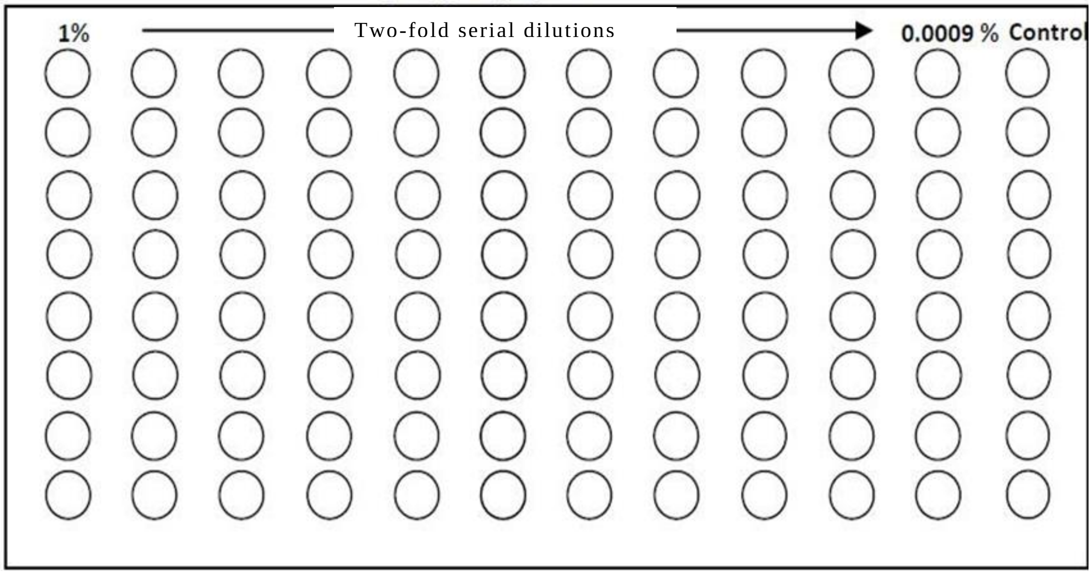

Figure S5: Example MIC plate for *S*. Typhi, wild-type, Δ*tolC*, and Δ*tolC*+ strains

Table.S2: MICs of antibiotics and detergents for the Δ*tolC*, Δ*tolC*+, and compared to wild-type reference strain.

**Genotype**		**MIC (µg/mL)**			
		**SDS**	**Bile**	**Chl**	**Tet**
ST-Wild-type		625	>3000	>4	>2
ST-Δ*tolC*	*tolC*; Kan^r	**39**	**156.25**	**1**	**0.5**
ST-Δ*tolC*+	pKK-*tolC*	625	>3000	>4	>2

Chl, chloramphenicol; Tet, tetracycline; SDS, sodium dodecyl sulfate. The value in boldface is smaller than those of the wild-type reference as a control strain. MIC determinations were repeated at list three times.
